# Risk of incident macular neovascularization in eyes with focal choroidal excavation: A retrospective cohort study with time-to-event analysis

**DOI:** 10.1371/journal.pone.0362252

**Published:** 2026-11-13

**Authors:** Marie Kitano, Tatsuya Inoue, Shuichiro Aoki, Shinji Kakihara, Kohdai Kitamoto, Ryo Terao, Ryo Obata, Keiko Azuma

**Affiliations:** 1 Department of Ophthalmology, The University of Tokyo, Bunkyo-ku, Tokyo, Japan; 2 Department of Ophthalmology, JR Tokyo General Hospital, Shibuya-ku, Tokyo, Japan; 3 Department of Ophthalmology and Micro-Technology, School of Medicine, Yokohama City University, Yokohama, Kanagawa, Japan; 4 Department of Ophthalmology, Toranomon Hospital, Minato-ku, Tokyo, Japan; Akita University: Akita Daigaku, JAPAN

## Abstract

**Purpose:**

To evaluate the long-term risk of incident macular neovascularization (MNV) in eyes with focal choroidal excavation (FCE) compared with non-FCE pachychoroid-spectrum controls in routine clinical practice.

**Methods:**

This retrospective cohort study included eyes with pachychoroid-spectrum disease and no evidence of MNV at baseline that were evaluated at the University of Tokyo Hospital between January 2008 and March 2025. Eyes were classified according to the presence or absence of optical coherence tomography-defined FCE. Eyes without FCE, including those with central serous chorioretinopathy and pachychoroid pigment epitheliopathy, served as the non-FCE control group. MNV-free survival was assessed using the Kaplan–Meier method, and hazard ratios (HRs) were estimated using an age-adjusted Cox proportional hazards model. An exploratory within-FCE analysis evaluated serous retinal detachment (SRD) involving the excavation site on baseline OCT.

**Results:**

A total of 608 eyes were included in the analysis, comprising 54 eyes with FCE and 554 non-FCE control eyes. Among the control eyes, 515 were classified as central serous chorioretinopathy and 39 as pachychoroid pigment epitheliopathy. During follow-up, incident MNV developed in 31 eyes overall, including 6 eyes in the FCE group and 25 eyes in the control group. The median follow-up period was 3.0 [interquartile range, 2.0–9.0] years in the FCE group and 3.0 [interquartile range, 1.0–7.0] years in the control group. In the age-adjusted Cox proportional hazards model, increasing age was associated with a higher hazard of incident MNV (HR 1.04 per year; 95% confidence interval: 1.01–1.07; *p* = 0.0133), and FCE was associated with a higher hazard of incident MNV compared with the non-FCE control group after adjustment for age (HR 2.74; 95% confidence interval: 1.003–6.387; p = 0.0298). In an exploratory within-FCE analysis, incident MNV developed in 5 of 13 eyes with SRD involving the excavation site at baseline, compared with 1 of 41 eyes without baseline SRD (Fisher’s exact test, p = 0.0021).

**Conclusions:**

In this real-world cohort of eyes with pachychoroid-spectrum disease, FCE was associated with a higher age-adjusted hazard of incident MNV compared with non-FCE controls. SRD involving the excavation site was more frequently observed among FCE eyes that developed incident MNV; however, this exploratory observation should be interpreted as hypothesis-generating.

## Introduction

Pachychoroid-spectrum diseases comprise a continuum of phenotypes characterized by increased choroidal thickness, dilated outer choroidal vessels (pachyvessels), and choroidal vascular hyperpermeability. These entities include pachychoroid pigment epitheliopathy (PPE), central serous chorioretinopathy (CSC), focal choroidal excavation (FCE), and pachychoroid neovasculopathy [1]. CSC is clinically significant because chronic or recurrent serous retinal detachment (SRD) can result in sustained stress and dysfunction of the outer retina and retinal pigment epithelium (RPE). In a subset of eyes, this process may subsequently lead to the development of macular neovascularization (MNV), which can cause irreversible visual loss [1–4]. Recent cohort studies have provided a more detailed characterization of CSC eyes that develop MNV [5–12]. In particular, the increasing use of optical coherence tomography angiography (OCTA) has enabled the detection of subclinical and early-stage MNV in real-world clinical settings and has facilitated the identification of associated clinical and imaging characteristics [5–12].

On optical coherence tomography (OCT), FCE is characterized by a localized choroidal concavity, typically involving the macular region [13]. FCE frequently coexists with other pachychoroid phenotypes and has been reported in CSC cohorts, supporting the concept of a shared pathophysiological spectrum [14,15]. Although many eyes with FCE remain stable without exudative changes, neovascular complications have been documented, including case series describing MNV complicating FCE [16,17]. However, compared with CSC, the long-term risk of incident MNV in eyes with FCE remains incompletely characterized, particularly in routine clinical practice settings in which follow-up intervals, censoring, and treatment exposure vary [1].

An additional practical challenge is that MNV may be clinically subtle at onset. The growing use of OCTA for the detection of subclinical or early MNV in pachychoroid-related diseases, including CSC, highlights the need for rigorous time-to-event definitions and follow-up characterization when assessing the risk of incident MNV [5].

Given these uncertainties, we hypothesized that eyes with FCE may have a distinct long-term risk of incident MNV compared with other non-FCE pachychoroid phenotypes encountered in routine clinical practice. Therefore, a retrospective cohort study was conducted using time-to-event analysis to compare incident MNV between eyes with FCE and non-FCE controls (CSC + PPE), accounting for age as a key covariate.

## Methods

### Study design and setting

This retrospective cohort study adhered to the tenets of the Declaration of Helsinki and was approved by the Institutional Review Board of the University of Tokyo (approval no. 2217). Because this was a retrospective chart-review study, the requirement for written informed consent was waived by the Institutional Review Board. Patients were provided the opportunity to opt out via a publicly available notice. Medical records of eyes diagnosed with FCE, CSC, or PPE at the University of Tokyo Hospital between January 2008 and March 2025 were reviewed. Data were accessed for research purposes between 01/03/2026 and 07/06/2026. This study was reported in accordance with the Strengthening the Reporting of Observational Studies in Epidemiology (STROBE) guidelines.

### Participants and group definitions

The present cohort was based on our previously established institutional database of pachychoroid-spectrum diseases, which included consecutive patients initially evaluated at the University of Tokyo Hospital from January 2008 to March 2021 and has been reported previously [2]. For the present study, the database was re-reviewed and extended through March 2025 to identify eligible eyes diagnosed with FCE, CSC, or PPE. Eyes were then re-screened according to the current eligibility criteria, and eyes with pachychoroid neovasculopathy or any evidence of MNV at baseline were excluded.

We first identified eyes with pachychoroid-spectrum disease without evidence of MNV at baseline. The study population was restricted to eyes showing independent pachychoroid features on multimodal imaging, including increased choroidal thickness with dilated outer choroidal vessels (pachyvessels) on OCT and pachychoroid-associated RPE changes; when indocyanine green angiography was performed, choroidal vascular hyperpermeability was used as supportive evidence [1,2]. Eyes were then classified according to the presence or absence of OCT-defined FCE. FCE was diagnosed based on OCT findings of a localized choroidal concavity in the absence of posterior staphyloma or scleral ectasia, as previously described [13]. The FCE group was defined by the presence of OCT-defined FCE at baseline among eyes fulfilling independent pachychoroid criteria. Because FCE can coexist with CSC, neither a prior diagnosis of CSC nor a history of SRD was used as an exclusion criterion for the FCE group. A previously documented clinical diagnosis of CSC and the presence of SRD involving the FCE excavation site on baseline OCT were recorded as separate variables. Throughout the manuscript, SRD refers to neurosensory retinal detachment associated with subretinal fluid on OCT, whereas SRF refers specifically to the fluid component itself; “exudation” or “exudative changes” was used only as a broader term for activity-related findings associated with MNV. Thus, the primary comparison was based on the presence or absence of FCE within pachychoroid-spectrum eyes, rather than a comparison between mutually exclusive “pure FCE” and CSC/PPE entities. FCE status was determined by retrospective review of the OCT images obtained at baseline. Eyes were classified as having FCE only when focal choroidal excavation was identifiable on baseline OCT images; FCE status was not assigned based on lesions first detected on images obtained later during follow-up. The non-FCE control group consisted of eyes without FCE that were diagnosed with CSC or PPE. CSC was defined as eyes with a history of SRD, while PPE was defined as pachychoroid eyes without a history of SRD. SRD history and exudative status were recorded separately. Eyes with pachychoroid neovasculopathy, polypoidal choroidal vasculopathy/aneurysmal type 1 neovascularization, or any evidence of MNV at baseline, including quiescent MNV detected on available baseline OCTA, were excluded from the analysis. Eyes were also excluded if they had other potential causes of MNV or conditions that could confound the diagnosis of pachychoroid-related MNV, including age-related macular degeneration with drusen, high or pathologic myopia, posterior staphyloma, inflammatory chorioretinal disease, inflammatory or myopic neovascularization, angioid streaks, ocular trauma, previous vitreoretinal surgery, substantial media opacity precluding reliable multimodal imaging assessment, or other macular disorders. Both eyes from the same patient were eligible for inclusion; to assess the potential influence of including both eyes from some patients, a left-eye-only sensitivity analysis was performed.

FCE/non-FCE classification was independently assessed by two retinal specialists using baseline OCT images. The graders were masked to subsequent development of incident MNV. Disagreements were resolved by consensus. Interobserver agreement for FCE classification was quantified using Cohen’s κ coefficient.

### Outcome and follow-up

The primary endpoint was incident MNV during follow-up. Baseline was defined as the first eligible visit at our institution at which the diagnosis of FCE, CSC, or PPE was established and the absence of MNV was confirmed on available baseline multimodal imaging. For eyes classified as FCE, the focal choroidal excavation was already identifiable on OCT at this baseline visit. Time-to-event was calculated from baseline to the first detection of MNV (event) or to censoring at the last available follow-up visit without evidence of MNV. Incident MNV was diagnosed on multimodal imaging, primarily OCT and OCTA, with fluorescein angiography and/or indocyanine green angiography used as adjunctive modalities when available. Eyes with evidence of MNV at baseline were excluded based on available multimodal imaging. Non-exudative (quiescent) MNV identified on baseline OCTA, when available, was also excluded from the analysis. OCTA was performed at the discretion of the treating physician, typically when MNV was clinically suspected or when exudative changes were suspected on OCT.

### Imaging acquisition and assessment

Structural OCT was performed as part of routine clinical care using a spectral-domain OCT device (SPECTRALIS OCT SHIFT; Heidelberg Engineering, Heidelberg, Germany; software version 2.5.9). Baseline structural OCT was required for eligibility assessment and FCE/non-FCE classification and was therefore available for all included eyes.

OCTA, when available, was performed using commercially available OCTA devices, including RTVue XR (Optovue, Fremont, CA, USA; software version 2016.2.0.35). OCTA scan protocols were selected according to clinical need, typically to evaluate suspected MNV or exudative changes, and were not fully standardized throughout the study period. Automated segmentation was reviewed by retinal specialists, and segmentation errors were manually corrected when necessary.

Because the study covered a long retrospective period from January 2008 to March 2025, OCTA was not available throughout the entire study period, and OCT/OCTA devices, scan protocols, and software versions were not fully standardized across the cohort.

Fluorescein angiography and indocyanine green angiography were performed when clinically indicated or when available at baseline as part of routine multimodal imaging assessment. The diagnosis of incident MNV was based on multimodal imaging findings, primarily structural OCT and OCTA, with FA and/or ICGA used as adjunctive modalities when available.

### Statistical analysis

Kaplan–Meier curves were generated to estimate MNV-free survival and were compared using the log-rank test, with the Wilcoxon test reported as a supportive analysis. Cox proportional hazards regression was used to estimate hazard ratios (HRs) and 95% confidence intervals (CIs), adjusting for age (per 1-year increase). In the primary Cox proportional hazards model, FCE status and age were included as covariates. Because photodynamic therapy (PDT) could be administered during follow-up, PDT exposure was summarized descriptively rather than incorporated as a fixed covariate in the primary Cox model. Baseline SRD at the excavation site was evaluated in an exploratory analysis restricted to eyes with FCE. SRD-positive eyes were defined as those in which SRD involving the FCE excavation site was present on baseline OCT. A previously documented diagnosis of CSC was recorded separately and was not used to determine baseline SRD status. SRD first observed after baseline or exudative fluid attributable to diagnosed MNV was not used to define baseline SRD positivity. Given the small number of MNV events in the FCE group, this analysis was considered exploratory and hypothesis-generating. CIs were computed using the profile likelihood method. Wald tests were used for hypothesis testing of individual covariates, whereas likelihood ratio tests were used to assess overall model fit. Two sensitivity analyses were performed: (1) restricting the control group to CSC-only (excluding PPE) and (2) a left-eye-only analysis to account for potential inter-eye correlation. The proportional hazards assumption was assessed and showed no major violations. Additional exploratory analyses were performed after revision. First, an exploratory analysis was restricted to eyes with a documented diagnosis of CSC to compare CSC eyes with FCE and those without FCE. Because the number of CSC-with-FCE eyes was small, this analysis was interpreted descriptively. Second, an exploratory Cox proportional hazards model additionally adjusted for central choroidal thickness was performed to assess whether the association between FCE and incident MNV was materially altered by differences in choroidal thickness. All analyses were performed using JMP software (version 16.0; SAS Institute, Cary, NC, USA). All statistical tests were two-sided, and a *p*-value of less than 0.05 was considered statistically significant. Continuous variables are presented as mean ± standard deviation or median [interquartile range], as appropriate. As an exploratory analysis within the FCE group, the association between SRD at the excavation site (SRD-positive *vs*. SRD-negative) and incident MNV was evaluated using Fisher’s exact test because of the small number of events.

## Results

A total of 608 eyes were included in the primary analysis, comprising 54 eyes with FCE and 554 non-FCE control eyes. Among the 54 eyes in the FCE group, all eyes fulfilled independent pachychoroid criteria. A previously documented clinical diagnosis of CSC was present in 11 FCE eyes, whereas the remaining 43 FCE eyes had pachychoroid features without a documented prior diagnosis of CSC. Among the non-FCE controls, 515 eyes were classified as CSC and 39 eyes as PPE. During follow-up, incident MNV developed in 31 eyes overall, including 6 eyes in the FCE group and 25 eyes in the control group. Baseline characteristics of the included eyes are summarized in Table 1. Interobserver agreement for FCE/non-FCE classification was excellent, with a Cohen’s κ coefficient of 0.971. Axial length, refractive error, and precise disease duration before baseline were not uniformly available in this retrospective referral-based cohort; therefore, these variables were not summarized in Table 1.

**Table 1:** Baseline characteristics of eyes included in the primary analysis.

Characteristic	FCE (*n* = 54)	Non-FCE controls (*n* = 554)	*P*-value*
No. of patients	52	464	—
Eyes per patient, *n* (%)			—
One eye	50 (96.2%)	374 (80.6%)	—
Two eyes	2 (3.8%)	90 (19.4%)	—
Laterality, *n* (%)			0.14
Right	32 (59.3%)	265 (47.8%)	
Left	22 (40.7%)	289 (52.2%)	
Diagnosis within non-FCE controls, *n* (%)	—	CSC: 515 (93.0%) PPE: 39 (7.0%)	—
Age, years (mean ± SD)	50.9 ± 14.5	55.6 ± 13.2	0.025
Male sex, *n* (%)	32 (59.3%)	399 (72.0%)	0.059
Follow-up, years, median [IQR]†	3.0 [2.0–9.0]	3.0 [1.0–7.0]	0.11
Incident MNV events, *n* (%)	6 (11.1%)	25 (4.5%)	—
Baseline BCVA, logMAR, mean ± SD	0.01 ± 0.14	0.06 ± 0.24	0.0231
Central choroidal thickness, µm, mean ± SD	263.1 ± 83.8	334.4 ± 98.6	<0.0001
Baseline SRD, n (%)	13 (24.1%)	515 (93.0%)	<0.0001
Documented CSC diagnosis, n (%)	11 (20.4%)	515 (93.0%)	<0.0001
Previous treatment before baseline, n (%)	2 (3.7%)	139 (25.1%)	0.00008
Incidence rate, per 1,000 eye-years‡	20.37	10.10	—

*P-values were calculated using Welch’s t-test for age, baseline BCVA, and central choroidal thickness; Fisher’s exact test for sex, baseline SRD, documented CSC diagnosis, and previous treatment before baseline; the chi-square test for laterality; and the Wilcoxon rank-sum test for follow-up duration.

†IQR, interquartile range.

‡Incidence rate was calculated as the number of events per 1,000 eye-years.

The availability of multimodal imaging and follow-up intensity are summarized in S1 Table. Baseline structural OCT was available in all included eyes. Baseline OCTA was available in 33 of 54 FCE eyes (61.1%) and 435 of 554 non-FCE control eyes (78.5%). Baseline FA and ICGA were available in 24 FCE eyes (44.4%) and 423 control eyes (76.4%). Follow-up OCTA before MNV detection or censoring was available in 45 FCE eyes (83.3%) and 446 control eyes (80.5%). The median number of visits before MNV detection or censoring was similar between groups: 15.5 [IQR, 5.0–31.8] in the FCE group and 16.0 [IQR, 5.0–31.0] in the control group.

Treatment exposure according to study group and timing is summarized in S2 Table. PDT before baseline was performed in 0 FCE eyes and 4 non-FCE control eyes. PDT between baseline and MNV detection or censoring was performed in 2 FCE eyes and 117 non-FCE control eyes. Among eyes that developed incident MNV, PDT before MNV detection had been performed in 2 of 6 FCE eyes and 10 of 25 non-FCE control eyes. PDT after MNV detection was performed in 0 FCE eyes and 5 non-FCE control eyes. Anti-VEGF therapy before baseline or before MNV detection/censoring was not performed in either group. After MNV detection, anti-VEGF therapy was administered in all 31 eyes that developed MNV, including 6 FCE eyes and 25 non-FCE control eyes.

Kaplan–Meier analysis demonstrated lower MNV-free survival in eyes with FCE than in non-FCE control eyes, although the difference did not reach statistical significance according to the log-rank test (*p* = 0.0693). In contrast, the Wilcoxon test, which places greater weight on earlier events, demonstrated a significant difference between the groups (*p* = 0.0078) (Fig 1).

**Fig 1:**
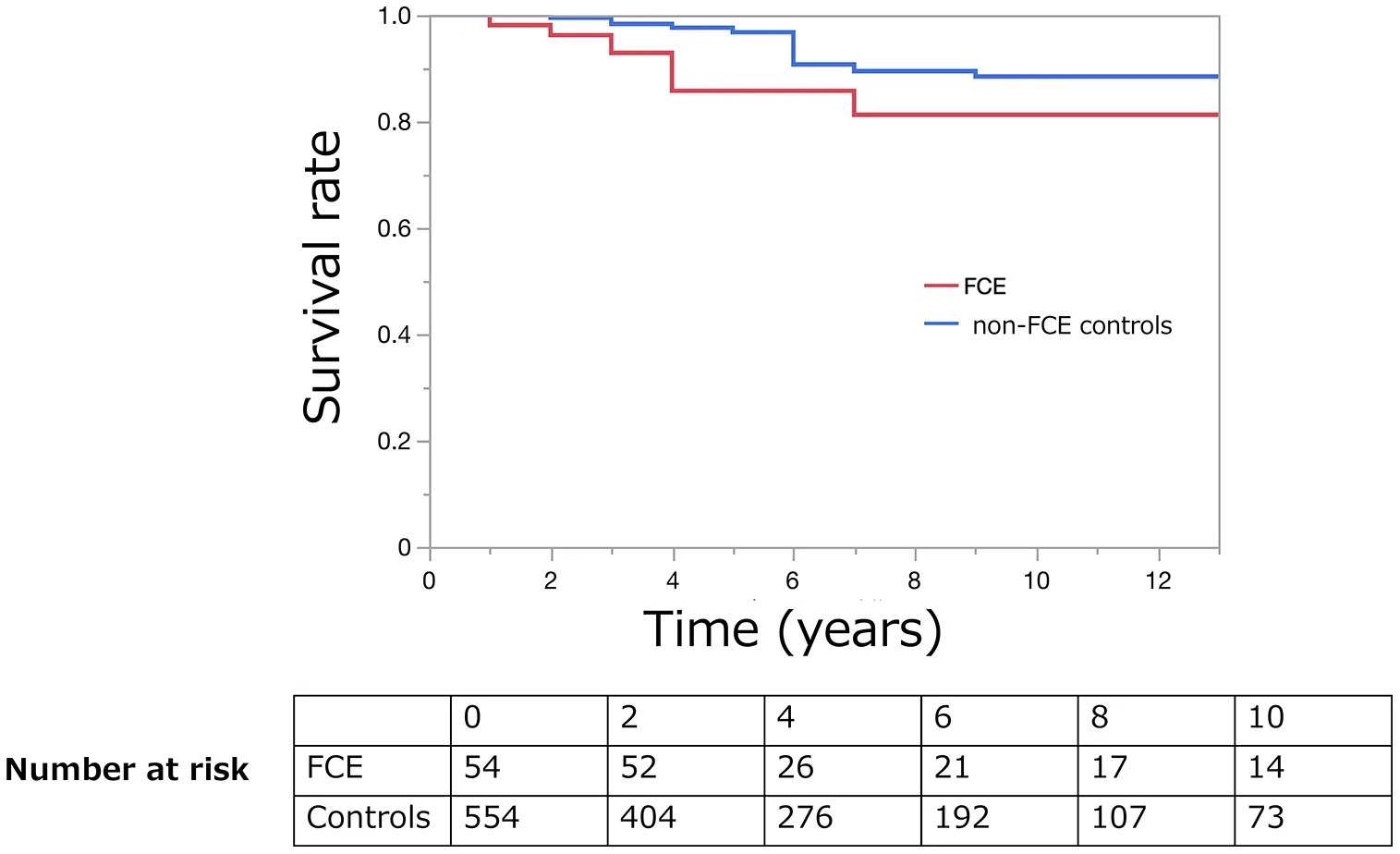
Kaplan–Meier curves for incident macular neovascularization (MNV) according to the presence of focal choroidal excavation. Kaplan–Meier curves showing the cumulative probability of remaining free from incident macular neovascularization in eyes with focal choroidal excavation (FCE) and non-FCE control eyes (CSC + PPE). The numbers at risk at baseline were 54 eyes in the FCE group and 554 eyes in the non-FCE control group. The log-rank test demonstrated a borderline difference between the groups (p = 0.0693), whereas the Wilcoxon test showed a significant difference (p = 0.0078).

In the primary age-adjusted Cox proportional hazards model, increasing age was associated with a higher hazard of incident MNV (HR 1.04 per year; 95% CI: 1.01–1.07; *p* = 0.0133). Compared with non-FCE controls, eyes with FCE exhibited a higher hazard of incident MNV after adjustment for age (HR 2.74; 95% CI: 1.003–6.387; Wald *p* = 0.0298) (Table 2).

**Table 2:** Age-adjusted Cox proportional hazards model for incident MNV.

Variable	HR	95% CI	*P*-value (Wald)
Age (per 1-year increase)	1.04	1.01–1.07	0.0133
FCE (*vs*. non-FCE controls [CSC + PPE])	2.74	1.003–6.387	0.0298

Hazard ratios (HRs) were estimated using Cox proportional hazards regression with age included as a continuous covariate and modeled per 1-year increase. Confidence intervals were calculated using the profile likelihood method for the primary, CCT-adjusted, and left-eye-only Cox models. Wald confidence intervals were used for the sensitivity analysis restricting the non-FCE control group to CSC eyes.

Sensitivity analyses yielded directionally consistent findings. When the control group was restricted to eyes with CSC, the analysis included 569 eyes, comprising 54 eyes with FCE and 515 CSC control eyes. In this model, FCE remained associated with a higher hazard of incident MNV (HR 2.51; 95% CI: 1.01–6.22; *p* = 0.0478), and age remained a significant predictor (HR 1.04 per year; 95% CI: 1.01–1.07; *p* = 0.0141). This analysis included 311 left eyes, comprising 22 FCE eyes and 289 non-FCE control eyes. Incident MNV developed in 5 FCE eyes and 14 non-FCE control eyes. In the age-adjusted Cox model, FCE remained associated with a higher hazard of incident MNV (HR 5.21; 95% CI: 1.66–13.9; Wald p = 0.0018).

As an additional exploratory sensitivity analysis restricted to eyes with a documented diagnosis of CSC, 526 eyes were analyzed, comprising 11 CSC-with-FCE eyes and 515 CSC-without-FCE eyes. Incident MNV developed in 4 of 11 CSC-with-FCE eyes and 25 of 515 CSC-without-FCE eyes. Because the number of CSC-with-FCE eyes was small, this analysis was considered exploratory. In an exploratory Cox model including age, FCE status, and central choroidal thickness, FCE remained associated with a higher hazard of incident MNV compared with non-FCE controls (HR 2.98; 95% profile-likelihood CI, 1.041–7.390; Wald p = 0.0267). Central choroidal thickness was not significantly associated with incident MNV (Wald p = 0.6131).

In an exploratory analysis restricted to the FCE group, SRD involving the excavation site was present on baseline OCT in 13 of 54 eyes and absent in 41 eyes. Incident MNV developed in 5 of 13 eyes with baseline SRD and in 1 of 41 eyes without baseline SRD (Fisher’s exact p = 0.0021) (Fig 2). Given the small number of events and potential residual confounding, this finding should be considered exploratory and hypothesis-generating.

**Fig 2:**
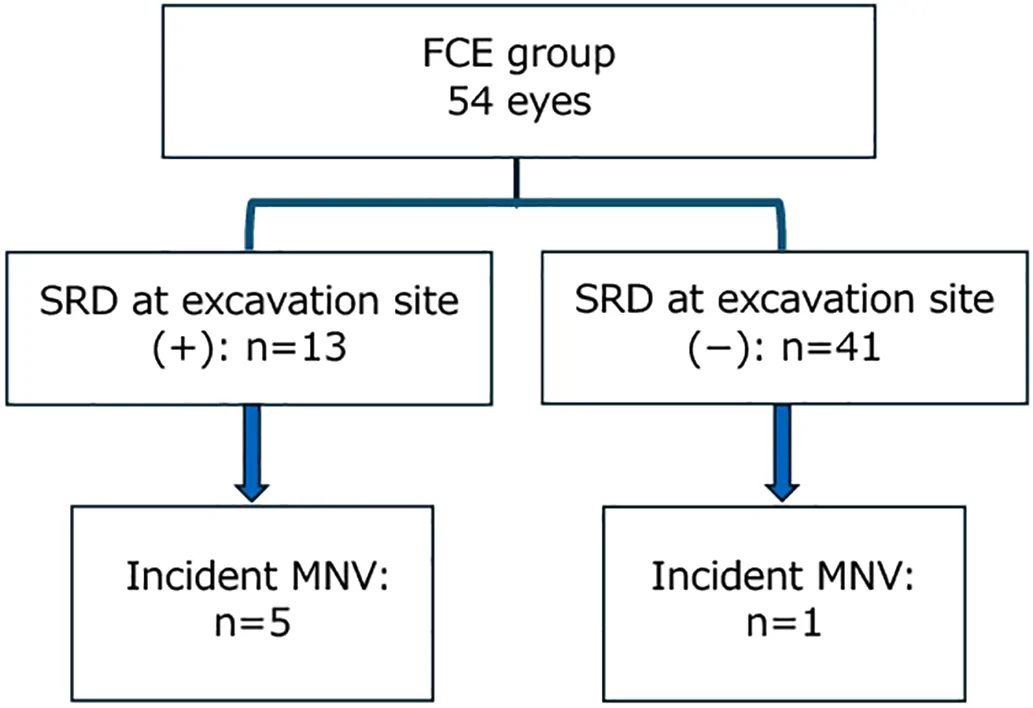
Exploratory analysis within the focal choroidal excavation (FCE) group according to the presence of baseline serous retinal detachment at the excavation site. Incident macular neovascularization (MNV) developed in 5 of 13 serous retinal detachment (SRD)-positive eyes at the excavation site and 1 of 41 SRD-negative eyes (Fisher’s exact *p* = 0.0021). Given the small number of events, this exploratory analysis should be considered hypothesis-generating.

Among the six eyes with FCE that developed incident MNV, two had undergone PDT before MNV detection. Among the control eyes with CSC that developed incident MNV, 10 had undergone PDT before MNV detection. Representative baseline and follow-up multimodal imaging findings illustrating the development of incident MNV at the site of FCE are shown in Figs 3 and 4, respectively.

**Fig 3:**
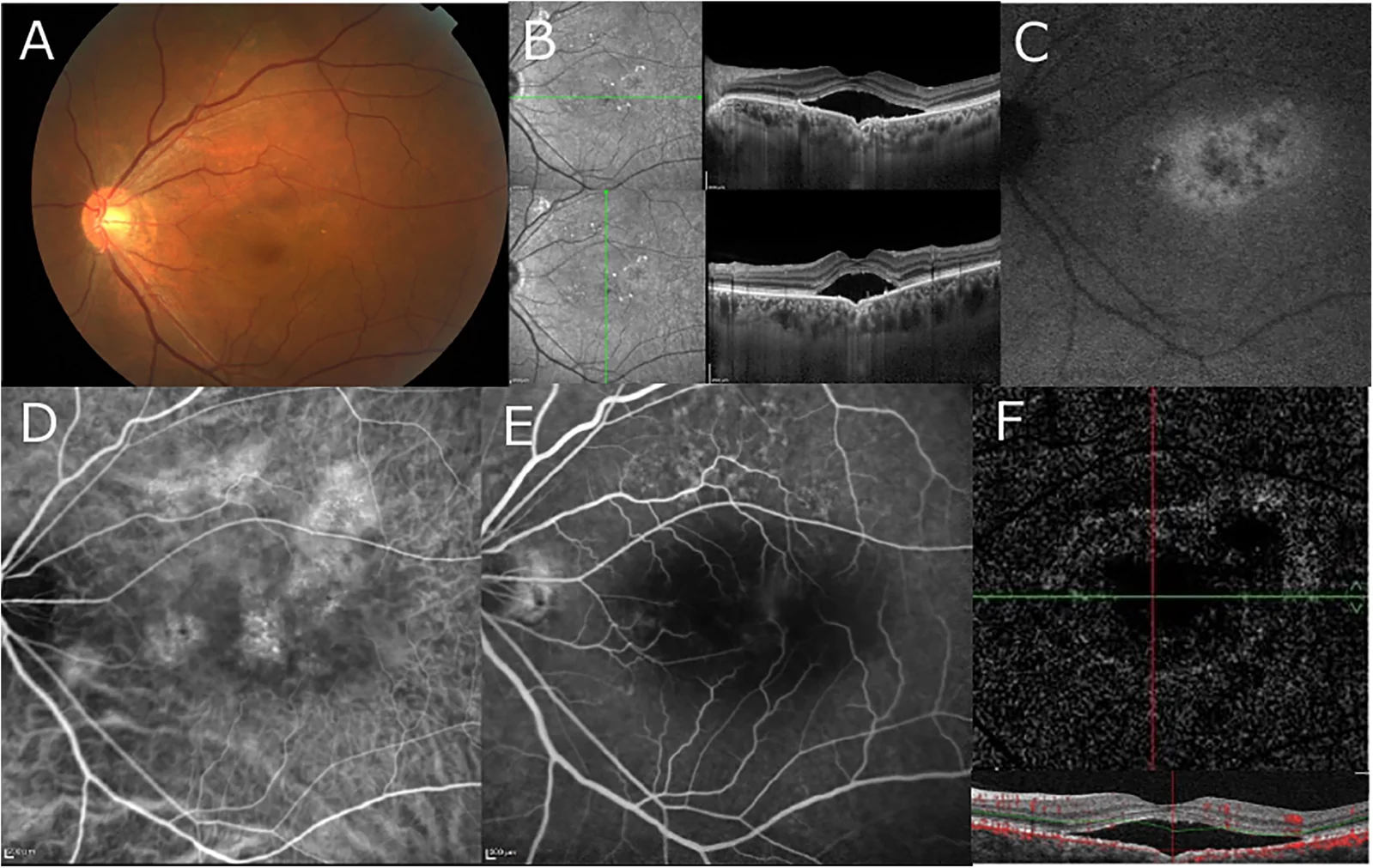
Baseline multimodal imaging of a representative eye with focal choroidal excavation that subsequently developed incident macular neovascularization. Baseline multimodal imaging findings of an eye with focal choroidal excavation (FCE) are shown. Color fundus photography demonstrated subtle pigmentary abnormalities at the macular region (A). Structural optical coherence tomography (OCT) demonstrated FCE with associated outer retinal and retinal pigment epithelium alterations and serous retinal detachment (SRD) (B). Fundus autofluorescence showed corresponding autofluorescence abnormalities at the lesion site (C). Fluorescein angiography and indocyanine green angiography demonstrated angiographic abnormalities corresponding to the FCE lesion, without definite evidence of macular neovascularization (MNV) (D, E). OCT angiography did not reveal a distinct neovascular flow signal (F).

**Fig 4:**
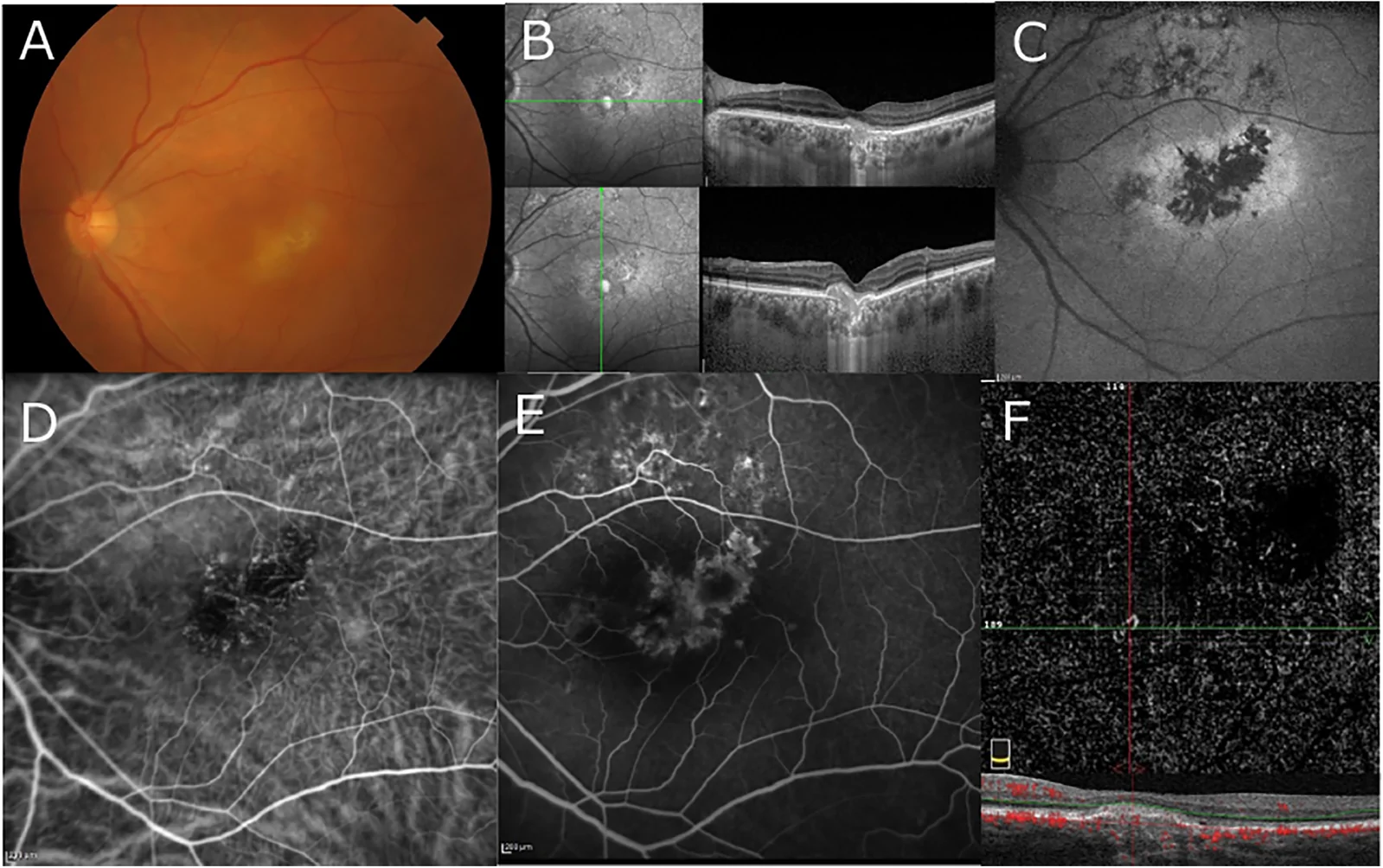
Follow-up multimodal imaging after the development of incident macular neovascularization in the same eye shown in Fig 3. Follow-up multimodal imaging obtained after the development of incident MNV is shown. The patient was followed for 11 years. SRD was present at the FCE site at baseline and resolved after photodynamic therapy. Incident MNV subsequently developed at the site of FCE 3.2 years after photodynamic therapy, accompanied by recurrent exudative changes. Color fundus photography demonstrated increased pigmentary and exudative changes surrounding the lesion (A). Structural OCT demonstrated exudative changes associated with excavation (B). Fundus autofluorescence showed enlargement of the retinal pigment epithelium abnormalities (C). Fluorescein angiography and indocyanine green angiography revealed findings consistent with active MNV (D, E). OCT angiography demonstrated a corresponding neovascular flow signal at the lesion site (F).

## Discussion

In this long-term retrospective cohort, eyes with FCE exhibited a higher age-adjusted hazard of incident MNV compared with non-FCE pachychoroid-spectrum controls (CSC + PPE) in time-to-event analyses. This finding is consistent with the current concept that pachychoroid-spectrum diseases include heterogeneous phenotypes with differing propensities for neovascular complications rather than a single spectrum with uniform risk [1,18].

A key consideration is the distinction between crude event proportions and hazard-based estimates. In routine clinical practice, follow-up duration and censoring are often heterogeneous; therefore, Kaplan–Meier and Cox regression provide a more robust assessment of time-dependent risk than simple cumulative proportions [1,5].

FCE is defined by OCT as a focal concavity of the choroid, and its clinical/OCT characteristics—comprising conforming and nonconforming configurations and overlap with other pachychoroid phenotypes—have been described [13]. Neovascular complications of FCE have been reported, including case series describing CNV/MNV arising at the base or slope of the excavation [16,17]. Our study extends previous reports by evaluating FCE within a comparative time-to-event framework against non-FCE pachychoroid controls encountered in routine practice and suggests that OCT-defined FCE was associated with an increased hazard of incident MNV in this cohort. Given the restricted number of events and retrospective ascertainment, the magnitude of the association should be interpreted cautiously; however, the direction of association was consistent across sensitivity analyses [1,19].

Age differed between groups and remained a significant covariate in the primary model, underscoring the importance of accounting for demographic imbalance when comparing pachychoroid phenotypes. This observation is consistent with clinical experience and previous studies demonstrating that age- and chronicity-related outer retinal/RPE alterations influence long-term outcomes in CSC-spectrum disease [1,2,4,20,21]. Although the association remained statistically significant after age adjustment, the number of MNV events in the FCE group was limited; therefore, the magnitude of the estimated effect should be interpreted cautiously, with emphasis on the direction of effect, internal consistency, and biological plausibility.

The direction of association (HR > 1 for FCE) was similar in the CSC-only and left-eye-only sensitivity analyses. However, given the limited number of MNV events, these sensitivity analyses should be interpreted as directionally consistent with the primary analysis rather than as confirmatory evidence.

An exploratory finding was that baseline SRD involving the FCE excavation site was more frequently observed among eyes that subsequently developed MNV. Although SRD status was determined at baseline, this association should be interpreted cautiously because of the small number of MNV events and potential residual confounding. Therefore, this finding remains hypothesis-generating rather than evidence that baseline SRD independently identifies a higher-risk FCE phenotype. Nevertheless, this finding is consistent with a conceptual model in which persistent SRD reflects RPE dysfunction and choroidal hyperpermeability/venous congestion, resulting in sustained outer retinal/RPE stress that may promote neovascular conversion in susceptible pachychoroid eyes [1,22].

The role of PDT should be interpreted cautiously in the present study. Among the six FCE eyes that developed incident MNV, two had undergone PDT before MNV detection; among CSC control eyes that developed incident MNV, 10 had undergone PDT before MNV detection. Although pre-event PDT may be clinically relevant, PDT exposure should be interpreted cautiously because it was performed according to clinical need and likely reflects disease activity, chronicity, and closer surveillance rather than a randomly assigned exposure. A formal time-dependent Cox sensitivity model with patient-level clustering was not performed because the number of incident MNV events, particularly in the FCE group, was limited and treatment timing was not standardized. Therefore, treatment exposure was summarized descriptively rather than modeled as a primary explanatory variable. These observations do not support a causal relationship between PDT and MNV development. Rather, prior PDT may serve as a surrogate marker of disease activity, chronicity, closer surveillance, or the possibility of pre-existing subclinical MNV [23–25] In addition, because PDT is frequently administered during follow-up, treating PDT exposure as a fixed baseline covariate in a conventional Cox model could introduce time-dependent bias. Future studies incorporating standardized OCTA surveillance and prespecified dates for SRD onset, PDT exposure, and MNV detection are needed to clarify the temporal relationship between treatment exposure and neovascular detection [5,6,26]. Strengths of this study include long-term real-world follow-up and a time-to-event analytic framework accounting for censoring. Limitations include the retrospective design, referral and treatment-selection biases, the limited number of MNV events in the FCE group, restricted precision of effect estimates, limited ability to assess effect modification, and the possibility of undetected subclinical neovascularization before the index date. OCTA is increasingly recognized as a valuable modality for detecting early/subclinical MNV in pachychoroid disorders, supporting standardized multimodal imaging in future studies [5,18,26,27]. Because both eyes from some patients were included, residual within-subject correlation remains a limitation; however, the left-eye-only sensitivity analysis yielded directionally consistent results. Another limitation is that time-dependent factors including SRD occurrence or recurrence and PDT exposure could not be formally assessed using time-dependent Cox models or landmark analysis approaches because the timing and duration of these exposures were not uniformly documented. Anti-VEGF treatment was administered after MNV detection and was therefore not considered a predictor of incident MNV. An important limitation is the non-standardized availability of OCTA and multimodal imaging during the long study period. OCTA was not available throughout the entire study period and was performed at the discretion of the treating physician, usually when MNV or exudative change was clinically suspected. Therefore, subclinical or quiescent MNV may have been underdetected, particularly in earlier cases and in eyes without baseline OCTA. This may have introduced surveillance or ascertainment bias. However, follow-up OCTA availability and the median number of visits before MNV detection or censoring were similar between the FCE and non-FCE control groups, suggesting that the observed association was not simply attributable to more intensive follow-up in the FCE group. These findings should nevertheless be interpreted as real-world time-to-event observations rather than estimates derived from a uniformly imaged prospective cohort.

These findings suggest the possibility that neovascular risk may differ among pachychoroid phenotypes, but they should not yet be used as definitive evidence for clinical risk stratification. In clinical practice, SRD at the excavation site may justify careful multimodal follow-up in selected FCE eyes, but standardized prospective studies are needed before specific surveillance strategies can be recommended. Larger multicenter cohorts, external validation studies, and standardized imaging protocols—particularly OCTA for the detection of subclinical MNV—will be important to validate these observations and improve risk models integrating structural phenotypes, exudative status, and treatment context [20,28].

## Conclusion

In this real-world retrospective cohort, eyes with FCE exhibited a higher age-adjusted hazard of incident MNV compared with non-FCE controls (CSC + PPE) in time-to-event analyses. However, given the small number of MNV events in the FCE group, the confidence interval close to the null, non-standardized baseline imaging, and possible residual confounding, this finding should be interpreted as a possible association rather than evidence that FCE is an independent risk factor for MNV. Larger multicenter studies incorporating standardized OCTA surveillance are warranted to validate these findings and further refine risk stratification.

## Supporting information

S1 Fig Participant flow diagram. Flow diagram showing the screening and inclusion of eyes from the institutional pachychoroid-spectrum disease database at the University of Tokyo Hospital between January 2008 and March 2025. Of 812 eyes screened, 204 were excluded because of baseline MNV or pachychoroid neovasculopathy, including polypoidal choroidal vasculopathy/aneurysmal type 1 neovascularization when present (n = 141), other potential causes of MNV or confounding macular disease (n = 18), insufficient baseline imaging (n = 11), insufficient follow-up information (n = 30), or other reasons (n = 4). The final analysis included 608 eyes: 54 eyes with FCE and 554 non-FCE controls, consisting of 515 eyes with CSC and 39 eyes with PPE. Abbreviations: CSC, central serous chorioretinopathy; FCE, focal choroidal excavation; MNV, macular neovascularization; PNV, pachychoroid neovasculopathy; PPE, pachychoroid pigment epitheliopathy.
(PNG)

S1 Table Multimodal imaging availability and follow-up intensity by group. (DOCX)

S2 Table Treatment exposure according to study group and timing. (DOCX)

S1 Dataset De-identified eye-level data underlying the study findings, with a variable dictionary. (XLSX)
