# Supplementary material for: Risk of incident macular neovascularization in eyes with focal choroidal excavation: A retrospective cohort study with time-to-event analysis
Source: PLoS One. 2026 Nov 13;21(11):e0362252. doi: 10.1371/journal.pone.0362252 (PMC13766242; doi:10.1371/journal.pone.0362252)
Supplement: Supplementary file 2 [file pone.0362252.s002.docx]

**Supplementary Table 1. Multimodal imaging availability and follow-up intensity by group**

| **Variable** | **FCE (n=54)** | **Non-FCE controls (n=554)** |
| --- | --- | --- |
| Baseline structural OCT | 54 (100%) | 554 (100%) |
| Baseline OCTA | 33 (61.1%) | 435 (78.5%) |
| Baseline FA | 24 (44.4%) | 423 (76.4%) |
| Baseline ICGA | 24 (44.4%) | 423 (76.4%) |
| Follow-up OCTA before MNV/censoring | 45 (83.3%) | 446 (80.5%) |
| Follow-up FA before MNV/censoring | 16 (29.6%) | 239 (43.1%) |
| Follow-up ICGA before MNV/censoring | 16 (29.6%) | 239 (43.1%) |
| No. of visits before MNV/censoring, median [IQR] | 15.5 [5.0–31.8] | 16.0 [5.0–31.0] |

**Abbreviations:** FA, fluorescein angiography; FCE, focal choroidal excavation; ICGA, indocyanine green angiography; IQR, interquartile range; MNV, macular neovascularization; OCT, optical coherence tomography; OCTA, optical coherence tomography angiography.

**Note:** Follow-up imaging indicates examinations performed before MNV detection or censoring.
