# Supplementary material for: Risk of incident macular neovascularization in eyes with focal choroidal excavation: A retrospective cohort study with time-to-event analysis
Source: PLoS One. 2026 Nov 13;21(11):e0362252. doi: 10.1371/journal.pone.0362252 (PMC13766242; doi:10.1371/journal.pone.0362252)
Supplement: Supplementary file 3 [file pone.0362252.s003.docx]

**Supplementary Table 2. Treatment exposure according to study group and timing**

| **Variable** | **FCE (n=54)** | **Non-FCE controls (n=554)** |
| --- | --- | --- |
| PDT before baseline | 0 eyes; 0 sessions | 4 eyes; 12 sessions |
| PDT between baseline and MNV detection/censoring | 2 eyes; 2 sessions | 117 eyes; 130 sessions |
| PDT after MNV detection | 0 eyes; 0 sessions | 5 eyes; 7 sessions |
| **Any PDT exposure** | **2 eyes; 2 sessions** | **124 eyes; 149 sessions** |
| Anti-VEGF before baseline | 0 eyes; 0 injections | 0 eyes; 0 injections |
| Anti-VEGF between baseline and MNV detection/censoring | 0 eyes; 0 injections | 0 eyes; 0 injections |
| Anti-VEGF after MNV detection | 6 eyes; 59 injections | 25 eyes; 505 injections |
| **Any anti-VEGF exposure** | **6 eyes; 59 injections** | **25 eyes; 505 injections** |

**Notes.** Values are presented as the number of treated eyes and the total number of treatment sessions or injections. PDT before MNV detection or censoring was mainly performed for persistent or recurrent SRD/CSC activity. PDT after MNV detection was performed as adjunctive treatment for MNV. Anti-VEGF therapy was administered after MNV detection and was therefore not considered a predictor of incident MNV. Treatment timing categories are not mutually exclusive; “any PDT exposure” indicates the number of unique eyes that received PDT at least once.

**Abbreviations.** CSC, central serous chorioretinopathy; FCE, focal choroidal excavation; MNV, macular neovascularization; PDT, photodynamic therapy; SRD, serous retinal detachment; VEGF, vascular endothelial growth factor.
