# Supplementary material for: Risk of incident macular neovascularization in eyes with focal choroidal excavation: A retrospective cohort study with time-to-event analysis
Source: PLoS One. 2026 Nov 13;21(11):e0362252. doi: 10.1371/journal.pone.0362252 (PMC13766242; doi:10.1371/journal.pone.0362252)
Supplement: Supplementary file 4 [file pone.0362252.s005.docx]

Dear Editor,

Thank you for the opportunity to review the manuscript entitled “Risk of Incident Macular Neovascularization in Eyes with Focal Choroidal Excavation: A Retrospective Cohort Study with Time-to-Event Analysis.”

This retrospective cohort study evaluates the long-term incidence of macular neovascularization (MNV) in 54 eyes with focal choroidal excavation (FCE) and 554 non-FCE pachychoroid-spectrum control eyes. The research question is clinically relevant, and the use of Kaplan–Meier and Cox proportional hazards analyses is appropriate for addressing heterogeneous follow-up and censoring. The long study period and comparative design are additional strengths.

However, several important methodological issues currently limit the interpretation of the reported association between FCE and incident MNV. In particular, the eligibility and diagnostic procedures require further clarification, OCTA surveillance was not standardized, treatment exposure is incompletely characterized, and the primary survival analysis does not adequately account for the inclusion of both eyes from some participants. The small number of MNV events in the FCE group also requires more cautious statistical interpretation.

My recommendation for this manuscript is **MAJOR REVISION**. You will find my detailed comments below.

Best regards,

- Participant selection and eligibility criteria: The inclusion and exclusion criteria are not described in sufficient detail to allow readers to reproduce the cohort or assess the possibility of selection bias. Were all consecutive patients diagnosed with FCE, CSC, or PPE during the study period screened? Please report the total number of potentially eligible eyes, the number excluded, and the reason for each exclusion, preferably in a participant flow diagram.
- The authors should clarify whether eyes with previous vitreoretinal surgery, high or pathologic myopia, posterior staphyloma, age-related macular degeneration or drusen, inflammatory or myopic neovascularization, other macular disorders, or substantial media opacity were excluded. The handling of polypoidal lesions/aneurysmal type 1 neovascularization should also be specified. Were eyes with pachychoroid neovasculopathy or other pachychoroid-spectrum diagnoses excluded at baseline because they already had MNV? Please provide explicit diagnostic and exclusion rules for these conditions. Also report who classified FCE/non-FCE status, whether grading was masked and independent, how disagreements were resolved, and interobserver agreement.
- OCTA was performed at the physician's discretion, and baseline quiescent MNV was excluded only when OCTA was available. Across the 2008-2025 period, this may have produced differential surveillance. Please report baseline and follow-up OCT/OCTA/angiography use and visit frequency by group.
- Please report, by group, how many eyes received PDT, the number of PDT sessions, anti-VEGF injections, indications, and whether treatment occurred before baseline, before MNV detection, or after MNV. If reliable dates are available, pre-event PDT may be assessed using a time-dependent Cox sensitivity model with patient-level clustering
- Table 1 should include baseline BCVA, axial length/refractive error, choroidal thickness, SRD status, disease duration, coexisting CSC, and previous treatment. Because FCE eyes were included regardless of CSC or SRD history, a CSC-with-FCE versus CSC-without-FCE sensitivity analysis would better isolate the association with FCE.
- Both eyes of some patients were included in the primary analysis, but it is unclear whether this was considered in the statistical evaluation. In addition, the right-eye-only analysis includes 311 eyes, whereas Table 1 reports only 297 right eyes. Please clarify and correct this numerical inconsistency and report the group sizes and number of MNV events in this analysis.
- Revise Table 1 so that IQR formatting is consistent and values such as 3.0 [2.0-9.0] remain on one line.
- In Figure 1, the blue curve is labeled “normal,” although this group consists of non-FCE eyes with CSC or PPE and should not be described as normal. Please replace “normal” with “non-FCE controls” or another accurate group label.
- Several passages use repetitive and formulaic wording. Language style alone cannot establish AI use; however, if generative AI supported drafting, translation, or language editing, please provide the journal-required disclosure, including the tool, its purpose, affected content, and how the output was verified.
