# Supplementary material for: Risk of incident macular neovascularization in eyes with focal choroidal excavation: A retrospective cohort study with time-to-event analysis
Source: PLoS One. 2026 Nov 13;21(11):e0362252. doi: 10.1371/journal.pone.0362252 (PMC13766242; doi:10.1371/journal.pone.0362252)
Supplement: Peer review history

# Peer review: Decision Letter 0

**DOI:** 10.1371/journal.pone.0362252.r001

**Authors:** Jiro Kogo

2 Oct 2026

Dear Dr. Azuma,

Please submit your revised manuscript by Nov 01 2026 11:59PM. If you will need more time than this to complete your revisions, please reply to this message or contact the journal office at plosone@plos.org. When you're ready to submit your revision, log on to https://www.editorialmanager.com/pone/ and select the 'Submissions Needing Revision' folder to locate your manuscript file.

- A letter that responds to each point raised by the academic editor and reviewer(s). You should upload this letter as a separate file labeled 'Response to Reviewers'.
- A marked-up copy of your manuscript that highlights changes made to the original version. You should upload this as a separate file labeled 'Revised Manuscript with Track Changes'.
- An unmarked version of your revised paper without tracked changes. You should upload this as a separate file labeled 'Manuscript'.

If you would like to make changes to your financial disclosure, please include your updated statement in your cover letter. Guidelines for resubmitting your figure files are available below the reviewer comments at the end of this letter.

If applicable, we recommend that you deposit your laboratory protocols in protocols.io to enhance the reproducibility of your results. Protocols.io assigns your protocol its own identifier (DOI) so that it can be cited independently in the future. For instructions see: https://journals.plos.org/plosone/s/submission-guidelines#loc-laboratory-protocols. Additionally, PLOS One offers an option for publishing peer-reviewed Lab Protocol articles, which describe protocols hosted on protocols.io. Read more information on sharing protocols at https://plos.org/protocols?utm_medium=editorial-email&utm_source=authorletters&utm_campaign=protocols.

As the corresponding author, your ORCID iD is verified in the submission system and will appear in the published article. PLOS supports the use of ORCID, and we encourage all coauthors to register for an ORCID iD and use it as well. Please encourage your coauthors to verify their ORCID iD within the submission system before final acceptance, as unverified ORCID iDs will not appear in the published article. *Only*  the individual author can complete the verification step; PLOS staff *cannot*  verify ORCID iDs on behalf of authors.

We look forward to receiving your revised manuscript.

Kind regards,

Jiro Kogo

Academic Editor

PLOS One

Journal Requirements:

When submitting your revision, we need you to address these additional requirements.

1. Please ensure that your manuscript meets PLOS ONE's style requirements, including those for file naming. The PLOS ONE style templates can be found at

https://journals.plos.org/plosone/s/file?id=wjVg/PLOSOne_formatting_sample_main_body.pdf and

https://journals.plos.org/plosone/s/file?id=ba62/PLOSOne_formatting_sample_title_authors_affiliations.pdf

2.  We note that you have indicated that there are restrictions to data sharing for this study. For studies involving human research participant data or other sensitive data, we encourage authors to share de-identified or anonymized data. However, when data cannot be publicly shared for ethical reasons, we allow authors to make their data sets available upon request. For information on unacceptable data access restrictions, please see http://journals.plos.org/plosone/s/data-availability#loc-unacceptable-data-access-restrictions.

Before we proceed with your manuscript, please address the following prompts:

a) If there are ethical or legal restrictions on sharing a de-identified data set, please explain them in detail (e.g., data contain potentially identifying or sensitive patient information, data are owned by a third-party organization, etc.) and who has imposed them (e.g., a Research Ethics Committee or Institutional Review Board, etc.). Please also provide contact information for a data access committee, ethics committee, or other institutional body to which data requests may be sent.

b) If there are no restrictions, please upload the minimal anonymized data set necessary to replicate your study findings to a stable, public repository and provide us with the relevant URLs, DOIs, or accession numbers. Please see http://www.bmj.com/content/340/bmj.c181.long for guidelines on how to de-identify and prepare clinical data for publication. For a list of recommended repositories, please see https://journals.plos.org/plosone/s/recommended-repositories. You also have the option of uploading the data as Supporting Information files, but we would recommend depositing data directly to a data repository if possible.

Please update your Data Availability statement in the submission form accordingly.

3. In the online submission form, you indicated that “De-identified data may be available from the corresponding author upon reasonable request and with approval from the Institutional Review Board of the University of Tokyo. Data requests may be directed to the corresponding author, Keiko Azuma, at keikoazuma719@gmail.com.”

All PLOS journals now require all data underlying the findings described in their manuscript to be freely available to other researchers, either 1. In a public repository, 2. Within the manuscript itself, or 3. Uploaded as supplementary information.

This policy applies to all data except where public deposition would breach compliance with the protocol approved by your research ethics board. If your data cannot be made publicly available for ethical or legal reasons (e.g., public availability would compromise patient privacy), please explain your reasons on resubmission and your exemption request will be escalated for approval.

4. Please include a caption for figure 4.

5. Please ensure that you refer to Figure 4 in your text as, if accepted, production will need this reference to link the reader to the figure.

6. If the reviewer comments include a recommendation to cite specific previously published works, please review and evaluate these publications to determine whether they are relevant and should be cited. There is no requirement to cite these works unless the editor has indicated otherwise.

7. Please review your reference list to ensure that it is complete and correct. If you have cited papers that have been retracted, please include the rationale for doing so in the manuscript text, or remove these references and replace them with relevant current references. Any changes to the reference list should be mentioned in the rebuttal letter that accompanies your revised manuscript. If you need to cite a retracted article, indicate the article’s retracted status in the References list and also include a citation and full reference for the retraction notice.

[Note: HTML markup is below. Please do not edit.]

Reviewers' comments:

Reviewer's Responses to Questions

**Comments to the Author**

1. Is the manuscript technically sound, and do the data support the conclusions?

Reviewer #1: Yes

Reviewer #2: Partly

**********

2. Has the statistical analysis been performed appropriately and rigorously?

Reviewer #1: Yes

Reviewer #2: Yes

**********

3. Have the authors made all data underlying the findings in their manuscript fully available?

The PLOS Data policy

Reviewer #1: Yes

Reviewer #2: Yes

**********

4. Is the manuscript presented in an intelligible fashion and written in standard English?

Reviewer #1: Yes

Reviewer #2: Yes

**********

Reviewer #1: Comments to the author

The authors report a retrospective cohort study evaluating incident MNV in eyes with FCE compared with non-FCE CSC/PPE controls. This is a clinically relevant research question and relatively longer follow-up period as well as use of time-to-event analysis are studies strengths. I have a few comments that may help clarify the methodology and ensure that the conclusions are appropriately supported by the data.

1. Report whether FCE status was determined from imaging at baseline or retrospectively from any image during follow-up. Also Clarify whether baseline was the first visit for the pachychoroid diagnosis, the first OCT demonstrating FCE, or the first visit at the institution.

2. FCE and control-group definitions need clarification. The manuscript should also explain whether every FCE eye was required to meet independent pachychoroid criteria. FCE is associated with pachychoroid but is not considered synonymous with a pachychoroid-spectrum diagnosis.

3. State whether eyes with other potential causes of MNV, such as AMD, high myopia, inflammatory disease, angioid streaks or trauma, were excluded.

4. The study spans January 2008 to March 2025. OCTA was not available throughout this period and baseline OCTA was performed only “when available.” OCTA during follow-up was also obtained at the treating physician’s discretion, usually when MNV was clinically suspected. The authors should report the OCT and OCTA devices used, scan protocols, segmentation methods, software versions and whether manual correction of segmentation errors was performed. Also provide proportions undergoing OCTA, FA and ICGA at baseline in each group; proportions undergoing OCTA during follow-up;imaging frequency or median number of visits per eye; an analysis restricted to the OCTA era or to eyes with adequate baseline multimodal imaging, if feasible

5. Please ensure consistent use of “serous retinal detachment,” “subretinal fluid” and “exudation,” since these are not necessarily interchangeable.

6. Clarify whether the representative case had baseline SRD continuously for 11 years, recurrent SRD or resolution after PDT. The temporal sequence is difficult to follow.

7. The SRD analysis has an unclear temporal definition and may be biased. The manuscript reports that 5/13 FCE eyes with SRD at the excavation site developed MNV compared with 1/41 without SRD. However, it is unclear whether “SRD-positive” refers to SRD present at baseline; any previous history of SRD; SRD occurring at any point during follow-up; or SRD present immediately before MNV diagnosis.

8. The interpretation by authors suggested is somewhat stronger than what is supported by the data.Only six MNV events occurred in the FCE group, the primary confidence interval is wide and close to the null, baseline MNV exclusion was not standardized and confounding is possible. Statements that the sensitivity analyses “support robustness” or that there is a “clinically meaningful difference” should therefore be moderated. The study supports a possible association that requires validation; it does not yet establish FCE as an independent risk factor, Therefore the stated conclusion needs to be revised.

9. Please reconcile the numbering and legends. The figure legends and submitted figure files have inconsistencies. The manuscript legend describes baseline and follow-up imaging as the upper and lower panels of Figure 3, whereas the submission contains separate files labelled Figures 3 and 4. Figure 4 does not appear to have a corresponding legend.

10. The authors refer to anti-VEGF treatment as a time-dependent factor but treatment occurs after MNV detection and would not ordinarily be a predictor of incident MNV. This sentence should be reconsidered.

Reviewer #2: Dear Editor,

Thank you for the opportunity to review the manuscript entitled “Risk of Incident Macular Neovascularization in Eyes with Focal Choroidal Excavation: A Retrospective Cohort Study with Time-to-Event Analysis.”

This retrospective cohort study evaluates the long-term incidence of macular neovascularization (MNV) in 54 eyes with focal choroidal excavation (FCE) and 554 non-FCE pachychoroid-spectrum control eyes. The research question is clinically relevant, and the use of Kaplan–Meier and Cox proportional hazards analyses is appropriate for addressing heterogeneous follow-up and censoring. The long study period and comparative design are additional strengths.

However, several important methodological issues currently limit the interpretation of the reported association between FCE and incident MNV. In particular, the eligibility and diagnostic procedures require further clarification, OCTA surveillance was not standardized, treatment exposure is incompletely characterized, and the primary survival analysis does not adequately account for the inclusion of both eyes from some participants. The small number of MNV events in the FCE group also requires more cautious statistical interpretation.

My recommendation for this manuscript is MAJOR REVISION. You will find my detailed comments below.

Best regards,

• Participant selection and eligibility criteria: The inclusion and exclusion criteria are not described in sufficient detail to allow readers to reproduce the cohort or assess the possibility of selection bias. Were all consecutive patients diagnosed with FCE, CSC, or PPE during the study period screened? Please report the total number of potentially eligible eyes, the number excluded, and the reason for each exclusion, preferably in a participant flow diagram.

• The authors should clarify whether eyes with previous vitreoretinal surgery, high or pathologic myopia, posterior staphyloma, age-related macular degeneration or drusen, inflammatory or myopic neovascularization, other macular disorders, or substantial media opacity were excluded. The handling of polypoidal lesions/aneurysmal type 1 neovascularization should also be specified. Were eyes with pachychoroid neovasculopathy or other pachychoroid-spectrum diagnoses excluded at baseline because they already had MNV? Please provide explicit diagnostic and exclusion rules for these conditions. Also report who classified FCE/non-FCE status, whether grading was masked and independent, how disagreements were resolved, and interobserver agreement.

• OCTA was performed at the physician's discretion, and baseline quiescent MNV was excluded only when OCTA was available. Across the 2008-2025 period, this may have produced differential surveillance. Please report baseline and follow-up OCT/OCTA/angiography use and visit frequency by group.

• Please report, by group, how many eyes received PDT, the number of PDT sessions, anti-VEGF injections, indications, and whether treatment occurred before baseline, before MNV detection, or after MNV. If reliable dates are available, pre-event PDT may be assessed using a time-dependent Cox sensitivity model with patient-level clustering

• Table 1 should include baseline BCVA, axial length/refractive error, choroidal thickness, SRD status, disease duration, coexisting CSC, and previous treatment. Because FCE eyes were included regardless of CSC or SRD history, a CSC-with-FCE versus CSC-without-FCE sensitivity analysis would better isolate the association with FCE.

• Both eyes of some patients were included in the primary analysis, but it is unclear whether this was considered in the statistical evaluation. In addition, the right-eye-only analysis includes 311 eyes, whereas Table 1 reports only 297 right eyes. Please clarify and correct this numerical inconsistency and report the group sizes and number of MNV events in this analysis.

• Revise Table 1 so that IQR formatting is consistent and values such as 3.0 [2.0-9.0] remain on one line.

• In Figure 1, the blue curve is labeled “normal,” although this group consists of non-FCE eyes with CSC or PPE and should not be described as normal. Please replace “normal” with “non-FCE controls” or another accurate group label.

• Several passages use repetitive and formulaic wording. Language style alone cannot establish AI use; however, if generative AI supported drafting, translation, or language editing, please provide the journal-required disclosure, including the tool, its purpose, affected content, and how the output was verified.

**********

what does this mean?). If published, this will include your full peer review and any attached files.

If you choose “no”, your identity will remain anonymous but your review may still be made public.

**Do you want your identity to be public for this peer review?**  For information about this choice, including consent withdrawal, please see our Privacy Policy

Reviewer #1: **Yes:**  Chinmayi Vyas

Reviewer #2: No

**********

[NOTE: If reviewer comments were submitted as an attachment file, they will be attached to this email and accessible via the submission site. Please log into your account, locate the manuscript record, and check for the action link "View Attachments". If this link does not appear, there are no attachment files.]

To ensure your figures meet our technical requirements, please review our figure guidelines: https://journals.plos.org/plosone/s/figures

You may also use PLOS’s free figure tool, NAAS, to help you prepare publication quality figures: https://journals.plos.org/plosone/s/figures#loc-tools-for-figure-preparation.

NAAS will assess whether your figures meet our technical requirements by comparing each figure against our figure specifications.

Attachment Submitted filename: PLOS ONE journal peer review.docx

# Author comment: Author response to Decision Letter 1

**DOI:** 10.1371/journal.pone.0362252.r002

16 Oct 2026

Response to the Editor and Reviewers

PLOS ONE

Manuscript title: Risk of Incident Macular Neovascularization in Eyes with Focal Choroidal Excavation: A Retrospective Cohort Study with Time-to-Event Analysis

Manuscript ID: PONE-D-26-30517

Dear Dr. Kogo and Reviewers,

We sincerely thank the Academic Editor and the reviewers for their careful evaluation of our manuscript and for the constructive comments. We have addressed each point in detail and revised the manuscript accordingly. Our point-by-point responses are provided below.

Page and line numbers in the responses below refer to the highlighted revised manuscript.

Reviewer 1

General comment:

The authors report a retrospective cohort study evaluating incident MNV in eyes with FCE compared with non-FCE CSC/PPE controls. This is a clinically relevant research question and relatively longer follow-up period as well as use of time-to-event analysis are studies strengths. I have a few comments that may help clarify the methodology and ensure that the conclusions are appropriately supported by the data.

#1. Comment: Report whether FCE status was determined from imaging at baseline or retrospectively from any image during follow-up. Also Clarify whether baseline was the first visit for the pachychoroid diagnosis, the first OCT demonstrating FCE, or the first visit at the institution.

Response: Thank you for this important comment. We agree that the timing of FCE classification and the definition of baseline required clarification. In the revised manuscript, we clarified that FCE status was determined by retrospective review of the OCT images obtained at baseline. FCE status was not assigned based on images first obtained later during follow-up. In all eyes classified as FCE, the focal choroidal excavation was identifiable on the baseline OCT images.

We also clarified that baseline was defined as the first eligible visit at our institution at which the diagnosis of FCE, CSC, or PPE was established, after confirming the absence of MNV on available baseline multimodal imaging. For eyes classified as FCE, this baseline visit corresponded to the visit at which FCE was already identifiable on OCT. Time-to-event was calculated from this baseline date to the first detection of incident MNV or to censoring at the last available follow-up visit.

Changes made: We revised the Participants and Group Definitions and Outcome and Follow-up sections (pages 5-7, lines 146-162 and 186-190).

#2. Comment: FCE and control-group definitions need clarification. The manuscript should also explain whether every FCE eye was required to meet independent pachychoroid criteria. FCE is associated with pachychoroid but is not considered synonymous with a pachychoroid-spectrum diagnosis.

Response: Thank you for this important comment. We agree that FCE should not be considered synonymous with pachychoroid-spectrum disease. We have clarified that all eyes in the FCE group fulfilled independent pachychoroid criteria on multimodal imaging, and that FCE status was defined by the presence of OCT-defined FCE at baseline. Because FCE may coexist with CSC in routine clinical practice, a history of CSC or SRD was not used as an exclusion criterion for the FCE group. A previously documented diagnosis of CSC was recorded separately from baseline SRD at the excavation site. Therefore, the primary comparison evaluated the presence versus absence of FCE among pachychoroid-spectrum eyes, rather than comparing mutually exclusive “pure FCE” and CSC/PPE entities.

Changes made: We revised the Participants and Group Definitions section and Results to clarify independent pachychoroid criteria, FCE/control definitions, and handling of documented CSC diagnosis (pages 5-6 and 9, lines 137-142, 146-162 and 266-270).

#3. Comment: State whether eyes with other potential causes of MNV, such as AMD, high myopia, inflammatory disease, angioid streaks or trauma, were excluded.

Response: Thank you for this important comment. We have clarified the exclusion criteria in the revised manuscript. Eyes with other potential causes of MNV were excluded, including age-related macular degeneration, high or pathologic myopia, posterior staphyloma, inflammatory chorioretinal disease, angioid streaks, ocular trauma, and other macular disorders that could confound the diagnosis of pachychoroid-related MNV. Eyes with pachychoroid neovasculopathy or any evidence of MNV at baseline, including quiescent MNV detected on available baseline OCTA, were also excluded. These exclusion criteria have been added to the Participants and Group Definitions section.

Changes made: We expanded the exclusion criteria in the Participants and Group Definitions section (page 6, lines 166-176).

#4. Comment: The study spans January 2008 to March 2025. OCTA was not available throughout this period and baseline OCTA was performed only “when available.” OCTA during follow-up was also obtained at the treating physician’s discretion, usually when MNV was clinically suspected. The authors should report the OCT and OCTA devices used, scan protocols, segmentation methods, software versions and whether manual correction of segmentation errors was performed. Also provide proportions undergoing OCTA, FA and ICGA at baseline in each group; proportions undergoing OCTA during follow-up;imaging frequency or median number of visits per eye; an analysis restricted to the OCTA era or to eyes with adequate baseline multimodal imaging, if feasible

Response: Thank you for this important comment. We agree that non-standardized OCTA availability and imaging surveillance over the long study period could have introduced differential surveillance or ascertainment bias.

We revised the Methods to describe the imaging procedures in greater detail, including the OCT and OCTA devices, software versions, segmentation review, and manual correction procedures. Structural OCT was performed using SPECTRALIS OCT SHIFT (Heidelberg Engineering; software version 2.5.9). OCTA was performed, when available, using RTVue XR (Optovue; software version 2016.2.0.35). OCTA scan protocols were selected according to clinical need and were not fully standardized throughout the retrospective study period. Automated segmentation was reviewed by retinal specialists, and segmentation errors were manually corrected when necessary.

We also added Supplementary Table 1 summarizing multimodal imaging availability and follow-up intensity by group. Baseline OCTA was available in 33 of 54 FCE eyes (61.1%) and 435 of 554 non-FCE control eyes (78.5%). Baseline FA and ICGA were available in 24 FCE eyes (44.4%) and 423 control eyes (76.4%). Follow-up OCTA before MNV detection or censoring was available in 45 FCE eyes (83.3%) and 446 control eyes (80.5%). The median number of visits before MNV detection or censoring was similar between groups: 15.5 [IQR, 5.0–31.8] in the FCE group and 16.0 [IQR, 5.0–31.0] in the control group.

We did not perform a formal OCTA-era-only analysis because OCTA availability was closely related to calendar time and clinical indication. Restricting the cohort to the OCTA era or to eyes with baseline OCTA could have introduced additional selection bias and would have reduced the number of events. Instead, we summarized imaging availability and visit frequency by group and expanded the Discussion to acknowledge the possibility of residual surveillance bias.

Changes made: We revised the Imaging acquisition and assessment section, Results, and Discussion, and added Supplementary Table 1 to summarize multimodal imaging availability and follow-up intensity (pages 7-9 and 15, lines 202-223, 278-286, and 446-457).

#5. Comment: Please ensure consistent use of “serous retinal detachment,” “subretinal fluid” and “exudation,” since these are not necessarily interchangeable.

Response: Thank you for this helpful comment. We agree that these terms should not be used interchangeably. We have reviewed the manuscript and revised the terminology for consistency. In the revised manuscript, “serous retinal detachment” (SRD) is used when referring to the clinical/OCT finding of neurosensory retinal detachment associated with subretinal fluid, particularly for the definition of CSC and for SRD at the FCE excavation site. “Subretinal fluid” (SRF) is used only when referring specifically to the fluid component on OCT. “Exudation” or “exudative changes” is used only as a broader term referring to activity-related findings, such as fluid or other exudative manifestations associated with MNV. We have revised the manuscript accordingly to avoid inconsistent terminology.

Changes made: We added terminology definitions in the Participants and Group Definitions section and revised wording throughout the manuscript for consistency (page 6, lines 146-162).

#6. Comment: Clarify whether the representative case had baseline SRD continuously for 11 years, recurrent SRD or resolution after PDT. The temporal sequence is difficult to follow.

Response: Thank you for this helpful comment. We agree that the temporal sequence in the representative case required clarification. We re-reviewed the clinical course and revised the Figure 4 legend to clarify the sequence of events. In this case, SRD was present at the FCE site at baseline and resolved after photodynamic therapy. Incident MNV subsequently developed at the site of FCE 3.2 years after photodynamic therapy, accompanied by recurrent exudative changes. Thus, the baseline SRD did not persist continuously throughout the 11-year follow-up period. We have revised the figure legend accordingly.

Changes made: We revised the Figure 4 legend to clarify the temporal sequence of SRD, PDT, and incident MNV in the representative case (page 25-26, lines 658-671).

#7. Comment: The SRD analysis has an unclear temporal definition and may be biased. The manuscript reports that 5/13 FCE eyes with SRD at the excavation site developed MNV compared with 1/41 without SRD. However, it is unclear whether “SRD-positive” refers to SRD present at baseline; any previous history of SRD; SRD occurring at any point during follow-up; or SRD present immediately before MNV diagnosis.

Response: Thank you for this important comment. We agree that the temporal definition of SRD at the excavation site required clarification. We confirmed that all 13 FCE eyes classified as SRD-positive had SRD involving the excavation site on baseline OCT. Thus, SRD status was determined at baseline, rather than based on SRD occurring during follow-up. A previously documented diagnosis of CSC was recorded separately and was not used to define baseline SRD positivity.

Incident MNV developed in 5 of 13 eyes with baseline SRD and in 1 of 41 eyes without baseline SRD (Fisher’s exact p = 0.0021). We revised the Abstract, Methods, Results, Discussion, and Figure 2 legend to clarify this definition. Given the small number of MNV events and the possibility of residual confounding, this finding remains exploratory and hypothesis-generating.

Changes made: We clarified the baseline definition of SRD and distinguished it from a prior diagnosis of CSC in the Abstract (page 2-3, lines 49–73), Methods (pages 5–8, lines 146–149 and 231–241), Results (page 12, lines 336–351), Discussion (pages 13–14, lines 393–396), and Figure 2 legend (page 25, lines 625–633).

#8. Comment: The interpretation by authors suggested is somewhat stronger than what is supported by the data.Only six MNV events occurred in the FCE group, the primary confidence interval is wide and close to the null, baseline MNV exclusion was not standardized and confounding is possible. Statements that the sensitivity analyses “support robustness” or that there is a “clinically meaningful difference” should therefore be moderated. The study supports a possible association that requires validation; it does not yet establish FCE as an independent risk factor, Therefore the stated conclusion needs to be revised.

Response: Thank you for this important comment. We agree that the interpretation in the original manuscript was too strong given the small number of MNV events in the FCE group, the confidence interval close to the null, the non-standardized baseline imaging over the long retrospective study period, and the possibility of residual confounding.

We have revised the Abstract, Discussion, and Conclusion to moderate the interpretation. We no longer describe FCE as an independent risk factor for incident MNV. Instead, we state that FCE was associated with a higher age-adjusted hazard of incident MNV in this retrospective real-world cohort, and that this finding should be interpreted as a possible association requiring validation. We also revised statements regarding the sensitivity analyses from implying “robustness” to stating that the findings were directionally consistent with the primary analysis. In addition, we moderated the interpretation of the exploratory SRD analysis and clarified that this observation should be considered hypothesis-generating.

Changes made: We moderated the Abstract, Discussion, and Conclusion to state that the findings suggest a possible association requiring validation rather than an independent risk factor, and revised the sensitivity-analysis interpretation (pages 2-3, 13-14, and 16, lines 66-73, 393-403, and 472-476).

#9. Comment: Please reconcile the numbering and legends. The figure legends and submitted figure files have inconsistencies. The manuscript legend describes baseline and follow-up imaging as the upper and lower panels of Figure 3, whereas the submission contains separate files labelled Figures 3 and 4. Figure 4 does not appear to have a corresponding legend.

Response: Thank you for pointing this out. We have reconciled the figure numbering and legends. Because the baseline and follow-up multimodal images were submitted as separate figure files, we revised the manuscript so that Figure 3 presents the baseline multimodal imaging findings and Figure 4 presents the follow-up imaging findings after the development of incident MNV. We also added a corresponding Figure 4 legend and revised the text citation in the Results section to cite Figures 3 and 4, respectively.

Changes made: We reconciled the figure numbering and revised the text citation and Figure 3/4 legends (pages 12-13 and 25-26, lines 355-357 and 643-686).

#10. Comment: The authors refer to anti-VEGF treatment as a time-dependent factor but treatment occurs after MNV detection and would not ordinarily be a predictor of incident MNV. This sentence should be reconsidered.

Response: Thank you for this important comment. We agree that anti-VEGF treatment should not be described as a time-dependent predictor of incident MNV, because anti-VEGF therapy was administered after MNV detection. We have revised the Discussion accordingly. In the revised manuscript, SRD occurrence or recurrence and PDT exposure are described as time-dependent factors that could not be formally assessed, whereas anti-VEGF treatment is no longer listed as a predictor of incident MNV. We also clarified that anti-VEGF treatment was administered after MNV detection and was therefore not evaluated as a predictor of incident MNV.

Changes made: We revised the Discussion to clarify that anti-VEGF treatment was administered after MNV detection and was not evaluated as a predictor of incident MNV (pages 9-10 and 15, lines 287-297 and 439-446).

Reviewer 2

General comment: Thank you for the opportunity to review the manuscript entitled “Risk of Incident Macular Neovascularization in Eyes with Focal Choroidal Excavation: A Retrospective Cohort Study with Time-to-Event Analysis.”

This retrospective cohort study evaluates the long-term incidence of macular neovascularization (MNV) in 54 eyes with focal choroidal excavation (FCE) and 554 non-FCE pachychoroid-spectrum control eyes. The research question is clinically relevant, and the use of Kaplan–Meier and Cox proportional hazards analyses is appropriate for addressing heterogeneous follow-up and censoring. The long study period and comparative design are additional strengths.

However, several important methodological issues currently limit the interpretation of the reported association between FCE and incident MNV. In particular, the eligibility and diagnostic procedures require further clarification, OCTA surveillance was not standardized, treatment exposure is incompletely characterized, and the primary survival analysis does not adequately account for the inclusion of both eyes from some participants. The small number of MNV events in the FCE group also requires more cautious statistical interpretation.

My recommendation for this manuscript is MAJOR REVISION. You will find my detailed comments below.

Best regards,

#1. Comment: Participant selection and eligibility criteria: The inclusion and exclusion criteria are not described in sufficient detail to allow readers to reproduce the cohort or assess the possibility of selection bias. Were all consecutive patients diagnosed with FCE, CSC, or PPE during the study period screened? Please report the total number of potentially eligible eyes, the number excluded, and the reason for each exclusion, preferably in a participant flow diagram.

Response: Thank you for this important comment. We agree that the participant selection process and eligibility criteria required further clarification. The present cohort was based on our previously established institutional database of pachychoroid-spectrum diseases, which included consecutive patients initially evaluated at the University of Tokyo Hospital from January 2008 to March 2021 and has been reported previously. For the present study, this database was re-reviewed and extended through March 2025 to identify eligible eyes diagnosed with FCE, CSC, or PPE. Eyes were then re-screened according to the current eligibility criteria.

We revised the Methods to clarify the data source, inclusion criteria, and exclusion criteria. Eyes with pachychoroid neovasculopathy or any evidence of MNV at baseline, including quiescent MNV detected on available baseline OCTA, were excluded. Eyes with other potential causes of MNV or confounding macular disease, such as age-related macular degeneration, high or pathologic myopia, posterior staphyloma, inflammatory chorioretinal disease, angioid streaks, ocular trauma, or other macular disorders, were also excluded.

The final analysis included 608 eyes, comprising 54 eyes with FCE and 554 non-FCE control eyes, including 515 eyes with CSC and 39 eyes with PPE. We have also added a participant flow diagram as Supplementary Figure 1 to summarize the screening and inclusion process.

Changes made: We revised the Participants and Group Definitions section to describe the data source and screening process and added a participant flow diagram as Supplementary Figure 1 (pages 5 and 26, lines 128-135 and 673-686).

#2. Comment: The authors should clarify whether eyes with previous vitreoretinal surgery, high or pathologic myopia, posterior staphyloma, age-related macular degeneration or drusen, inflammatory or myopic neovascularization, other macular disorders, or substantial media opacity were excluded. The handling of polypoidal lesions/aneurysmal type 1 neovascularization should also be specified. Were eyes with pachychoroid neovasculopathy or other pachychoroid-spectrum diagnoses excluded at baseline because they already had MNV? Please provide explicit diagnostic and exclusion rules for these conditions. Also report who classified FCE/non-FCE status, whether grading was masked and independent, how disagreements were resolved, and interobserver agreement.

Response: Thank you for this important comment. We agree that the diagnostic and exclusion rules, as well as the image-grading process, required clarification. We have revised the Participants and Group Definitions section to explicitly state the exclusion criteria. Eyes with pachychoroid neovasculopathy, polypoidal choroidal vasculopathy/aneurysmal type 1 neovascularization, or any evidence of MNV at baseline, including quiescent MNV detected on available baseline OCTA, were excluded. We also clarified that eyes with other potential causes of MNV or conditions that could confound the diagnosis of pachychoroid-related MNV were excluded, including age-related macular degeneration with drusen, high or pathologic myopia, posterior staphyloma, inflammatory chorioretinal disease, inflammatory or myopic neovascularization, angioid streaks, ocular trauma, previous vitreoretinal surgery, substantial media opacity precluding reliable multimodal imaging assessment, and other macular disorders.

We further clarified the image-grading process. FCE/non-FCE classification was independently assessed by two retinal specialists using baseline OCT images. Disagreements were resolved by consensus. Interobserver agreement for FCE/non-FCE classification was excellent, with a Cohen’s κ coefficient of 0.971.

Changes made: We expanded the exclusion criteria and image-grading description in the Participants and Group Definitions section and reported interobserver agreement in the Results (pages 6 and 9, lines 166-183 and 273-277).

#3. Comment: OCTA was performed at the physician's discretion, and baseline quiescent MNV was excluded only when OCTA was available. Across the 2008-2025 period, this may have produced differential surveillance. Please report baseline and follow-up OCT/OCTA/angiography use and visit frequency by group.

Response: Thank you for this important comment. We agree that non-standardized OCTA availability and imaging surveillance over the long study period could have introduced differential surveillance or ascertainment bias.

We revised the Methods to describe the imaging procedures in greater detail, including the OCT and OCTA devices, software versions, segmentation review, and manual correction procedures. Structural OCT was performed using SPECTRALIS OCT SHIFT (Heidelberg Engineering; software version 2.5.9). OCTA was performed, when available, using RTVue XR (Optovue; software version 2016.2.0.35). OCTA scan protocols were selected according to clinical need and were not fully standardized throughout the retrospective study period. Automated segmentation was reviewed by retinal specialists, and segmentation errors were manually corrected when necessary.

We also added Supplementary Table 1 summarizing multimodal imaging availability and follow-up intensity by group. Baseline structural OCT was available for all included eyes. Baseline OCTA was available in 33 of 54 FCE eyes (61.1%) and 435 of 554 non-FCE control eyes (78.5%). Baseline FA and ICGA were available in 24 FCE eyes (44.4%) and 423 control eyes (76.4%). Follow-up OCTA before MNV detection or censoring was available in 45 FCE eyes (83.3%) and 446 control eyes (80.5%). The median number of visits before MNV detection or censoring was similar between groups: 15.5 [IQR, 5.0–31.8] in the FCE group and 16.0 [IQR, 5.0–31.0] in the non-FCE control group.

We did not perform a formal OCTA-era-only analysis because OCTA availability was closely related to calendar time and clinical indication. Restricting the cohort to the OCTA era or to eyes with baseline OCTA could have introduced additional selection bias and would have reduced the number of events. Instead, we summarized imaging availability and visit frequency by group and expanded the Discussion to acknowledge the possibility of residual surveillance bias.

Changes made: We revised the Imaging acquisition and assessment section, summarized imaging availability and visit frequency in the Results, added Supplementary Table 1, and expanded the Discussion on surveillance bias (pages 7-9 and 15, lines 202-223, 278-286, and 443-457).

#4. Comment: Please report, by group, how many eyes received PDT, the number of PDT sessions, anti-VEGF injections, indications, and whether treatment occurred before baseline, before MNV detection, or after MNV. If reliable dates are available, pre-event PDT may be assessed using a time-dependent Cox sensitivity model with patient-level clustering.

Response: Thank you for this important comment. We agree that treatment exposure should be described more clearly according to study group and timing. We have added Supplementary Table 2 summarizing PDT and anti-VEGF exposure in the FCE and non-FCE control groups. For each treatment, we recorded the number of treated eyes, number of PDT sessions or anti-VEGF injections, treatment indication, and whether treatment occurred before baseline, between baseline and MNV detection or censoring, or after MNV detection.

In the FCE group, PDT was performed in 2 eyes between baseline and MNV detection or censoring, with a total of 2 PDT sessions. No FCE eyes received PDT before baseline or after MNV detection. In the non-FCE control group, PDT was performed in 4 eyes before baseline, 117 eyes between baseline and MNV detection or censoring, and 5 eyes after MNV detection, with 149 PDT sessions in total. PDT before MNV detection or censoring was mainly performed for persistent or recurrent SRD/CSC activity, whereas PDT after MNV detection was performed as adjunctive treatment for MNV.

Anti-VEGF therapy was not administered before baseline or before MNV detection/censoring in either group. After MNV detection, anti-VEGF therapy was administered in all 31 eyes that developed MNV, including 6 FCE eyes and 25 non-FCE control eyes.

We did not perform a formal time-dependent Cox sensitivity model with patient-level clustering because the number of incident MNV events, particularly in the FCE group, was limited and treatment timing was not standardized. In addition, PDT exposure was clinically driven and likely reflected disease activity, chronicity, and surveillance intensity. We therefore summarized treatment exposure descriptively and expanded the Discussion to acknowledge this limitation.

Changes made: We added Supplementary Table 2 and revised the Results and Discussion to describe treatment exposure by group and timing, including the rationale for not performing a time-dependent Cox model (pages 9-10 and 14-15, lines 287-297 and 411-439).

#5. Comment: Table 1 should include baseline BCVA, axial length/refractive error, choroidal thickness, SRD status, disease duration, coexisting CSC, and previous treatment. Because FCE eyes were included regardless of CSC or SRD history, a CSC-with-FCE versus CSC-without-FCE sensitivity analysis would better isolate the association with FCE.

Response: Thank you for this important suggestion. We agree that additional baseline characteristics would help readers assess potential differences between the FCE and non-FCE control groups. We therefore expanded Table 1 to include baseline BCVA, central choroidal thickness, baseline SRD status, documented CSC diagnosis or coexisting CSC, and previous treatment before baseline.

Axial length, refractive error, and precise disease duration before baseline were not uniformly available in this retrospective referral-based cohort. Therefore, these variables could not be reliably summarized in Table 1 or included in the primary Cox model. However, eyes with high or pathologic myopia and posterior staphyloma were excluded according to the eligibility criteria, as clarified in the revised Methods.

We also performed an additional exploratory sensitivity analysis restricted to eyes with a documented diagnosis of CSC, to better isolate the association between FCE and incident MNV among eyes with CSC features. This analysis included 526 eyes, comprising 11 CSC-with-FCE eyes and 515 CSC-without-FCE eyes. Incident MNV developed in 4 of 11 CSC-with-FCE eyes and 25 of 515 CSC-without-FCE eyes. Because the number of CSC-with-FCE eyes was small, this analysis was considered exploratory and interpreted cautiously.

In addition, because central choroidal thickness differed between groups, we performed an exploratory Cox model additionally adjusted for age and central choroidal thickness. In an exploratory Cox model including age, FCE status, and central choroidal thickness, FCE remained associated with a higher hazard of incident MNV compared with non-FCE controls (HR 2.98; 95% profile-likelihood CI, 1.041–7.390; Wald p = 0.0267). Central choroidal thickness was not significantly associated with incident MNV (Wald p = 0.6131).

Changes made: We expanded Table 1, added a note on unavailable axial length, refractive error, and disease duration, added exploratory analyses in the Statistical Analysis section, and reported the documented CSC diagnosis–restricted and CCT-adjusted analyses in the Results (pages 8-12, lines 247-255, 273-277, 313-317, and 336-345).

#6. Comment: Both eyes of some patients were included in the primary analysis, but it is unclear whether this was considered in the statistical evaluation. In addition, the right-eye-only analysis includes 311 eyes, whereas Table 1 reports only 297 right eyes. Please clarify and correct this numerical inconsistency and report the group sizes and number of MNV events in this analysis.

Response: Thank you for pointing out this important inconsistency. We apologize for the error. The sensitivity analysis was incorrectly described as a right-eye-only analysis in the original manuscript. The analysis actually included left eyes only. We have corrected this throughout the revised manuscript.

In the corrected left-eye-only sensitivity analysis, 311 eyes were included, comprising 22 FCE eyes and 289 non-FCE control eyes. Incident MNV developed in 5 FCE eyes and 14 non-FCE control eyes. After adjustment for age, FCE remained associated with a higher hazard of incident MNV (HR 5.21; 95% CI: 1.66–13.9; Wald p = 0.0018).

Changes made: We corrected the right-eye-only analysis to a left-eye-only sensitivity analysis and reported the group sizes and number of MNV events in the Methods and Results (pages 6, 8, and 10, lines 177-178, 245-247, and 313-317).

#7. Comment: Revise Table 1 so that IQR formatting is consistent and values such as 3.0 [2.0-9.0] remain on one line.

Response: Thank you for pointing this out. We revised Table 1 to ensure consistent formatting of median and interquartile range values. All values are now presented in the format median [IQR], using a consistent en dash for the IQR range. We also shortened the column headings and adjusted the table layout so that values such as 3.0 [2.0–9.0] remain on a single line.

Changes made: We reformatted Table 1 to use consistent median [IQR] formatting and shortened headings to avoid line breaks (pages 10-11, Table 1, lines 320-324).

#8. Comment: In Figure 1, the blue curve is labeled “normal,” although this group consists of non-FCE eyes with CSC or PPE and should not be described as normal. Please replace “normal” with “non-FCE controls” or another accurate group label.

Response: Thank you for pointing this out. We agree that the term “normal” was inaccurate, because the comparison group consisted of non-FCE pachychoroid-spectrum eyes with CSC or PPE rather than normal eyes. We have revised Figure 1 accordingly, replacing “normal” with “non-FCE controls.” The figure legend has also been revised for consistency.

Changes made: We revised Figure 1 to label the blue curve as 'non-FCE controls' and updated the Figure 1 legend accordingly (page 25, lines 625-633).

#9. Comment: Several passages use repetitive and formulaic wording. Language style alone cannot establish AI use; however, if generative AI supported drafting, translation, or language editing, please provide the journal-required disclosure, including the tool, its purpose, affected content, and how the output was verified.

Response: Thank you for this comment. We have revised the manuscript to reduce repetitive and formulaic wording. We also added an AI use disclosure statement to the revised manuscript.

During the revision process, the authors used ChatGPT (OpenAI) to assist with English language editing, translation of reviewer comments, and refinement of wording in the revised manuscript and response to reviewers. The tool was not used to generate research hypotheses, collect data, perform statistical analyses, create or alter primary research data, diagnose cases, or generate study results. All AI-assisted text was critically reviewed, edited, and verified by the authors against the original clinical data, statistical outputs, imaging findings, and relevant literature. The authors take full responsibility for the accuracy, integrity, and final content of the manuscript.

Changes made: We added the AI use disclosure statement to the manuscript (pages 23-24, lines 616-623).

Attachment Submitted filename: renamed_cab04.pdf

# Editor report: Decision Letter 1

**DOI:** 10.1371/journal.pone.0362252.r003

**Authors:** Jiro Kogo

22 Oct 2026

Risk of Incident Macular Neovascularization in Eyes with Focal Choroidal Excavation: A Retrospective Cohort Study with Time-to-Event Analysis

PONE-D-26-30517R1

Dear Dr. Azuma

We’re pleased to inform you that your manuscript has been judged scientifically suitable for publication and will be formally accepted for publication once it meets all outstanding technical requirements.

Within one week, you’ll receive an e-mail detailing the required amendments. When these have been addressed, you’ll receive a formal acceptance letter and your manuscript will be scheduled for publication.

An invoice will be generated when your article is formally accepted. Please note, if your institution has a publishing partnership with PLOS and your article meets the relevant criteria, all or part of your publication costs will be covered. Please make sure your user information is up-to-date by logging into Editorial Manager at Editorial Manager® and clicking the ‘Update My Information' link at the top of the page. For questions related to billing, please contact billing support.

If your institution or institutions have a press office, please notify them about your upcoming paper to help maximize its impact. If they’ll be preparing press materials, please inform our press team as soon as possible -- no later than 48 hours after receiving the formal acceptance. Your manuscript will remain under strict press embargo until 2 pm Eastern Time on the date of publication. For more information, please contact onepress@plos.org.

Kind regards,

Jiro Kogo

Academic Editor

PLOS One

Additional Editor Comments (optional):

Reviewers' comments:

# Editor report: Acceptance letter

**DOI:** 10.1371/journal.pone.0362252.r004

**Authors:** Jiro Kogo

PONE-D-26-30517R1

PLOS One

Dear Dr. Azuma,

I'm pleased to inform you that your manuscript has been deemed suitable for publication in PLOS One. Congratulations! Your manuscript is now being handed over to our production team.

At this stage, our production department will prepare your paper for publication. This includes ensuring the following:

* All references, tables, and figures are properly cited

* All relevant supporting information is included in the manuscript submission,

* There are no issues that prevent the paper from being properly typeset

You will receive further instructions from the production team, including instructions on how to review your proof when it is ready. Please keep in mind that we are working through a large volume of accepted articles, so please give us a few days to review your paper and let you know the next and final steps.

Lastly, if your institution or institutions have a press office, please let them know about your upcoming paper now to help maximize its impact. If they'll be preparing press materials, please inform our press team within the next 48 hours. Your manuscript will remain under strict press embargo until 2 pm Eastern Time on the date of publication. For more information, please contact onepress@plos.org.

You will receive an invoice from PLOS for your publication fee after your manuscript has reached the completed accept phase. If you receive an email requesting payment before acceptance or for any other service, this may be a phishing scheme. Learn how to identify phishing emails and protect your accounts at https://explore.plos.org/phishing.

If we can help with anything else, please email us at customercare@plos.org.

Thank you for submitting your work to PLOS One and supporting open access.

Kind regards,

PLOS One Editorial Office Staff

on behalf of

Prof. Jiro Kogo

Academic Editor

PLOS One
